# P67 Learning points from a service evaluation exploring diagnosis and management of urinary tract infection with bacteraemia caused by Gram negative bacilli in Northwest London

**DOI:** 10.1093/jacamr/dlaf230.074

**Published:** 2025-12-04

**Authors:** Francesca Siracusa, Megha Anil, Nafisa Badat, Nikita Acharya, Grace Gysin, Selina Salim, Amarachi Ohanusi, Priya Khanna, Guduru Gopal Rao, Amit Amin, Ashley Whittington

**Affiliations:** Northwick Park Hospital, London North West University Healthcare NHS Trust, London, UK; Northwick Park Hospital, London North West University Healthcare NHS Trust, London, UK; Northwick Park Hospital, London North West University Healthcare NHS Trust, London, UK; Northwick Park Hospital, London North West University Healthcare NHS Trust, London, UK; Northwick Park Hospital, London North West University Healthcare NHS Trust, London, UK; Northwick Park Hospital, London North West University Healthcare NHS Trust, London, UK; Faculty of Medicine, Imperial College London, London, UK; Department of Clinical Microbiology and Antimicrobial Stewardship, London North West University Healthcare NHS Trust, London, UK; Department of Clinical Microbiology and Antimicrobial Stewardship, London North West University Healthcare NHS Trust, London, UK; Department of Clinical Microbiology and Antimicrobial Stewardship, London North West University Healthcare NHS Trust, London, UK; Department of Infectious Diseases, Northwick Park Hospital, London North West University Healthcare NHS Trust, London, UK

## Abstract

**Objectives:**

To evaluate diagnosis and management of patients with Gram-negative bacilli (GNB) bacteraemia with urinary source to improve patient care and antimicrobial stewardship in our NHS Trust.

**Methods:**

We randomly selected adult patients from a Northwest London NHS Trust’s National Mandatory Surveillance Database from April 2024 to March 2025. Patients were included if the source was confirmed or suspected as urinary tract. Clinical and outcome data were extracted from the electronic patient record (Cerner) and Laboratory Information Management System (Helix).

**Results:**

In total, 105 patients were included; median age was 62 years, and 66/105 (62.9%) were female. Overall, 28/105 (27.7%) were ethnically Indian, 15/105 (14.9%) ‘Other Asian’ and 15/105 (14.9%) Non-British White. 65/105 presented with fever (61.9%), 37/105 had urinary symptoms (35.2%) and 15/105 had delirium (14.3%). 53/105 (50.5%) reported other symptoms including dizziness, breathlessness, vomiting, abdominal pain, or anorexia. 44/105 (41.9%) had type 2 diabetes, 9/105 (8.6%) had a long-term catheter and 10/105 (9.5%) had a urinary tract structural abnormality/prosthesis. 15/105 (15.2%) of patients received antibiotics in the community, but over two-thirds were ineffective due to resistance. 94/105 (89.5%) of bloodstream infections were *Escherichia coli* followed by 7/105 (6.7%) *Pseudomonas aeruginosa*. 35/105 (33.3%) were fully sensitive and 27/105 (25.7%) were ESBLs. 17/27 (63.0%) of patients with ESBL bacteraemia were ethnically Asian. Urine samples most frequently grew no organism (41/91, 45.1%). 31/50 (62.0%) positive urine cultures grew *E. coli* and 7/50 (14.0%) grew *Pseudomonas*. The admitting medical team most frequently prescribed co-amoxiclav and amikacin. 79.0% of patients were on an effective regular antibiotic at the time of positive blood culture. Median length of stay was 9 days. 17/105 (16.2%) were re-admitted within 30 days and 5/105 (4.8%) died within this period. 4/5 deaths were during the index admission, 2/5 died from sepsis, 2/5 from metastatic cancer and 1/5 suddenly without clear cause. Outcomes were similar between patients managed by infectious diseases and internal medicine.

**Conclusions:**

The ethnic diversity of our sample reflects the population living in our Trust catchment area. Presenting complaints were varied, reflecting the diagnostic uncertainty at presentation. Type 2 diabetes was common in this patient group (40%). Most patients treated in the community who required admission received ineffective antibiotic treatment in the community due to AMR. Despite high ESBL rates (25.7%), case-fatality rate was far below national averages. Two-thirds of those with ESBL bacteraemia were of Asian ethnicity.

**Take-home points:**

Clinicians should consider urinary source GNB bacteraemia in unwell older patients with risk factors, even in the absence of fever or urinary symptoms. Type 2 diabetes was more prevalent than expected in this patient group and further study is warranted to explore this as a risk factor. More work is needed to assist GPs in identifying patients at highest risk of ESBL bacteraemia so that appropriate and timely antibiotic therapy can be started, ideally prior to clinical deterioration. ESBL rates were high (25.7%) and ethnically Asian patients were disproportionally represented, which is in keeping with the findings of previous studies in Northwest London.

